# Prevention of bisphosphonate-related osteonecrosis of the jaw with basic fibroblast growth factor: an experimental study in rats

**DOI:** 10.1007/s10266-025-01073-w

**Published:** 2025-02-20

**Authors:** Satoshi Kurokawa, Takahiro Yagyuu, Naoki Funayama, Mitsuhiko Imada, Tadaaki Kirita

**Affiliations:** Department of Oral and Maxillofacial Surgery, Nara Medical University, Kashihara-Shi, Nara Japan

**Keywords:** Bisphosphonate, Osteonecrosis, Basic fibroblast growth factor, Tooth extraction, Angiogenesis

## Abstract

Bisphosphonate-related osteonecrosis of the jaw (BRONJ), characterized by persistent jaw bone exposure, is believed to result from the inhibition of bone remodeling and wound healing, often associated with potent intravenous bisphosphonates such as zoledronic acid (ZA). These bisphosphonates are known to significantly impact the healing of soft and bone tissues due to their anti-angiogenic properties. Our study aimed to explore whether applying the basic fibroblast growth factor (bFGF), known for its ability to promote angiogenesis and bone remodeling, over extraction sockets could prevent the onset of BRONJ in rats administered with ZA. The experimental protocol involved administering each rat with two intravenous injections of 60 μg/kg of ZA, followed by tooth extraction. Rats were then divided into two groups: the test group (*n* = 12), which had a mixture of 3% hydroxypropyl cellulose (HPC) and 0.3% bFGF applied over their extraction sockets, and the control group (*n* = 12), which received only a 3% HPC application. Wound healing was monitored for 8 weeks post-extraction and assessed using micro-computed tomography, histological evaluations, and immunohistochemical analysis of CD31- and CD105-positive vessels. Results showed a BRONJ incidence rate of 100% (12/12) in the control group, whereas the rate was significantly lower in the test group at 8.3% (1/12). Furthermore, the test group demonstrated marked increases in both angiogenesis and new bone formation. Our findings suggest that the local application of bFGF might serve as an effective therapeutic approach to prevent the onset of BRONJ following tooth extraction in patients receiving bisphosphonate therapy.

## Introduction

Bisphosphonate-related osteonecrosis of the jaw (BRONJ) is characterized by exposure of the bone in the lower and upper jaws that persists for more than 8 weeks, typically without prior radiation therapy to the jawbone or metastasis of malignant tumors from other sites [1]. Bisphosphonates (BPs), known for their inhibition of angiogenesis and bone remodeling, have been widely and safely utilized to effectively treat conditions like malignant hypercalcemia, osteoporosis, bone metastasis of solid cancers, and multiple myeloma [2–5]. However, BRONJ is thought to result from the inhibition of neovascularization and bone remodeling. Zoledronic acid (ZA), a highly potent intravenously used BP, significantly affects soft tissue and osseous healing due to its anti-angiogenic action [6–9]. It has been reported that 63.7% of BRONJ cases are associated with tooth extraction [10]. Clinical studies on BRONJ prevention have suggested that perioperative antibiotic prophylaxis and primary wound closure can often prevent BRONJ, though these preventive methods are not always successful [11–16].

Basic fibroblast growth factor (bFGF), which promotes neovascularization and bone remodeling [17–20], is clinically used to treat intractable skin ulcers, tympanic perforation, and alveolar bone defects caused by periodontitis [20–24]. Thus, this study aims to determine whether the application of bFGF to the extraction sockets of ZA-administered rats could prevent the onset of BRONJ.

## Materials and methods

### Creation of a rat BRONJ model

This animal study was approved by the animal care and use committee of Nara Medical University (Permit Number: 12997). A total of 24 female Sprague–Dawley (SD) rats (Japan SLC, Shizuoka, Japan), aged 10 weeks, were used in this study. The rats were kept in an environment with a controlled temperature and 12-h light–dark cycles, with food and water supplied ad libitum. Surgery was performed under general anesthesia, and all efforts were made to minimize suffering.

This study used a rat BRONJ model, similar to that used in a previous study [25]. All rats received two intravenous injections of 60 μg/kg of ZA, based on the human dosage of 4 mg/65.8 kg. The first dose was administered at the beginning of the experiment after imaging. The second dose was given 1 week after the first dose, on the date of the molar extraction. The intravenous ZA injections were administered into the tail vein, which was revealed by the application of a rubber band tourniquet. After induction of anesthesia with a single intraperitoneal injection of pentobarbital sodium 50 mg/kg, the left lower first molar was extracted from each rat. A gingival sulcus incision was made around the lower first molar using a no.11 surgical blade. A mucoperiosteal flap was raised using a periosteal elevator, and the molar was removed using a sharpened dental explorer. A 2-mm-diameter twist drill was used to expand the extraction socket to a standardized depth of 3 mm. Each extraction socket was expanded with a drill to create a standardized bone defect with a size of 2 × 2 × 3 mm.

The rats were randomly divided into the test group (*n* = 12) and the control group (*n* = 12), in which 3% hydroxypropyl cellulose (HPC) + 0.3% bFGF/12μL or 3% HPC/12μL, respectively, was applied over the defects. Finally, the defect was completely closed through the placement of a horizontal mattress suture using 5/0 polypropylene. The weight of the rats was measured at 2, 4, 6, and 8 weeks after tooth extraction. At the end of the experiment, the rats were euthanized and sacrificed by exsanguination under general anesthesia.

### Macroscopic analysis

Wound healing was assessed macroscopically at 2, 4, 6, and 8 weeks after tooth extraction. Mucosal healing was classified into three groups as follows: score 0, complete healing with normal mucosa covering; score 1, no clinical evidence of necrotic bone but nonspecific inflammatory findings at the extraction site; and score 2, exposed necrotic bone.

### Micro-computed tomography (CT) analysis

The harvested mandibles were analyzed using a micro-CT system. Each mandible was scanned at intervals of 38 μm at 90 kV and 88 μA. The finding in each alveolar defect was assessed on a coronal section 1.0-mm mesial from the mesial surface of the lower second molar. The extraction site was classified into three groups as follows: score 0, no sequestration; score 1, sequestration of the alveolar bone in the extraction socket; and score 2, sequestration of the entire alveolar ridge.

### Histological analysis and quantification of new bone formation

After micro-CT analysis, all mandibles were fixed in 10% formaldehyde neutral buffer solution, decalcified with 0.5 mol/l ethylenediaminetetraacetic acid (EDTA), and embedded in paraffin. Thin sections, 5 μm in thickness, were cut 1.0 mm mesial from the mesial surface of the lower second molar in the buccolingual direction and stained with hematoxylin and eosin (H&E) for a light microscopic examination. During the histological analysis, new bone formation was identified by the presence of a well-organized eosinophilic bone matrix that contained osteocytes within lacunae. The newly formed bone trabeculae appeared more cellular and vascularized compared to older, mature bone. In contrast, necrotic bone was characterized by the absence of osteocytes within the lacunae, presenting as empty lacunae. Mucosal disruption was assessed based on the continuity of the epithelial layer, with particular attention to any areas exhibiting ulceration or thinning of the mucosa. Sequestration was identified by the presence of devitalized bone fragments that had become detached from the surrounding viable bone.

These sections quantified the area of new bone formation in the extraction socket and were examined for the presence of mucosal disruption and sequestration. The areas of new bone formation in the extraction socket were quantified using BZ-Analyzer software (BZ-Analyzer, Keyence). Investigators blinded to the results of the micro-CT analyses and to the identification of the rat groups independently performed the histological analysis.

### Immunohistochemical analysis

Paraffin-embedded sections were stained using anti-CD31 antibody (sc-1506-R, Santa Cruz Biotechnology, Dallas, TX, USA) and anti-CD105 antibody (HPA011862, Sigma-Aldrich, St. Louis, USA) to evaluate the number of CD31- and CD105-positive vessels, a marker for neo-angiogenesis.

To ensure that the region of interest (ROI) was consistently and accurately identified in all samples, we standardized the procedure as follows:

The ROI was defined as a 500 × 300 μm rectangular area within the lamina propria at the site of the extraction socket. The upper boundary of the 500 μm-wide ROI was aligned with an imaginary line extending from the basement membrane of the adjacent normal mucosa. The midpoint of this upper boundary coincided with a line drawn across the middle of the upper edge of the alveolar bone at the extraction socket. From each end of this upper boundary, vertical lines were drawn downward for 300 μm to form the sides of the ROI. The lower boundary was then established by connecting the ends of these vertical lines, resulting in a rectangular area of 500 × 300 μm. This clearly defined and consistent ROI was applied across all samples to ensure the accuracy of comparisons between the test and control groups.

The images of immunohistochemically stained sections were captured using an all-in-one fluorescence BZ-X810 microscope (Keyence, Osaka, Japan). For each staining, all positive vessels within the defined ROI were counted using ImageJ software (National Institute of Health, USA). The total number of positive vessels per ROI was then used to statistically compare the expression levels between the groups.

### Statistical analysis

Nominal, ordinal, and continuous data were analyzed using the Chi-square, Mann–Whitney *U* test, and Student's *t* test, respectively. *P* < 0.01 denoted statistical significance. For statistical analyses, STATA data analysis and statistical software (version 14; StataCorp, College Station, TX, USA) was used [26].

## Results

### Changes in body weight

The mean body weight of the rats was 266.6 ± 9.6 g at the beginning of ZA administration, 275.4 ± 8.3 g after tooth extraction, and 290.4 ± 13.0 g at 8 weeks after tooth extraction. Thus, regardless of the treatment after tooth extraction, the body weight increased in all rats.

### Macroscopic findings

**Fig. 1:**
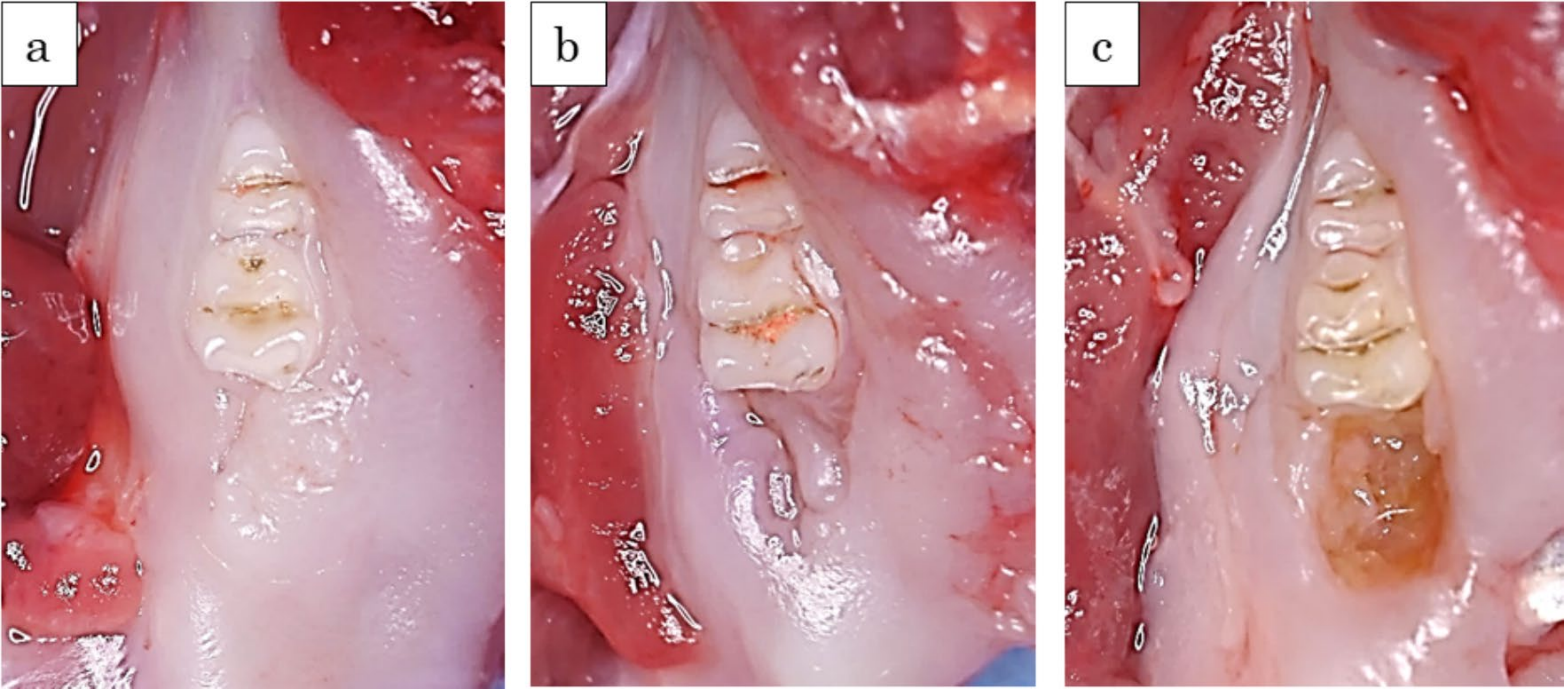
Typical macroscopic findings. **a** Extraction socket in the test group at 8 weeks after tooth extraction (score 0). The extraction socket was covered with normal mucosa, similar to the surrounding areas, and demonstrated no signs of inflammation. There was no residual socket concavity. **b** Extraction socket in the test group at 8 weeks after tooth extraction (score 1). The extraction socket was covered with normal mucosa, similar to the surrounding areas, and demonstrated no signs of inflammation, but the socket side was still concave. **c** Extraction socket in the control group at 8 weeks after tooth extraction (score 2). The extraction socket showed discolored, brownish, exposed bone

**Fig. 2:**
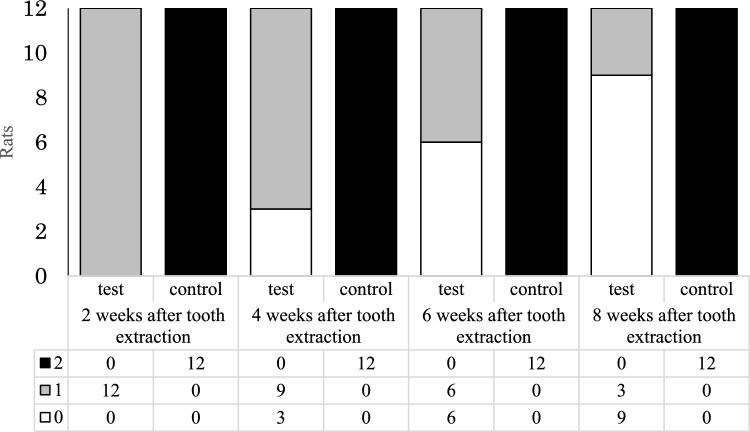
Score-based progression of macroscopic findings in extraction sockets over 8 weeks. This figure illustrates the evolving macroscopic observations in the extraction sockets for both the test and control groups over a period of 8 weeks post-tooth extraction. In the test group, all subjects (100%) were classified as score 1 after 2 weeks; this changed at 4 weeks, with a split of 25% score 0 and 75% score 1. By week 6, the test group had equal proportions of score 0 and score 1 (50% each), and at the 8-week mark, the majority of the test group (75%) had transitioned to score 0, while the remainder (25%) were still at score 1. On the other hand, throughout the entire 8-week observation period, all subjects in the control group consistently remained at score 2

Our macroscopic findings are depicted in Figs. 1 and 2, which present typical observations and the progression over time in both groups, respectively. Two weeks post-tooth extraction, the extraction socket in the test group was covered by normal mucosa, displaying no signs of inflammation, such as redness, and closely resembled the surrounding areas. However, the socket concavities persisted (score 1) in all the test subjects. In stark contrast, the sockets in the control group unanimously exposed bone (score 2).

Eight weeks following tooth extraction, a majority (75%) of the test group displayed sockets covered with normal mucosa, devoid of inflammatory changes or residual socket concavities (score 0) (Fig. 1a). In the remaining 25% of the test group, although the sockets were covered with normal mucosa, the socket side retained its concavity (score 1) (Fig. 1b). As for the control group, all subjects revealed discolored, brownish, exposed bone (score 2) in the sockets (Fig. 1c).

The osteonecrosis incidence at 8 weeks post-tooth extraction was starkly different between the two groups: 0% (0/12) in the test group and 100% (12/12) in the control group. This demonstrates a significantly higher incidence of osteonecrosis in the control group (*P* < 0.001).

### Micro-CT findings

**Fig. 3:**
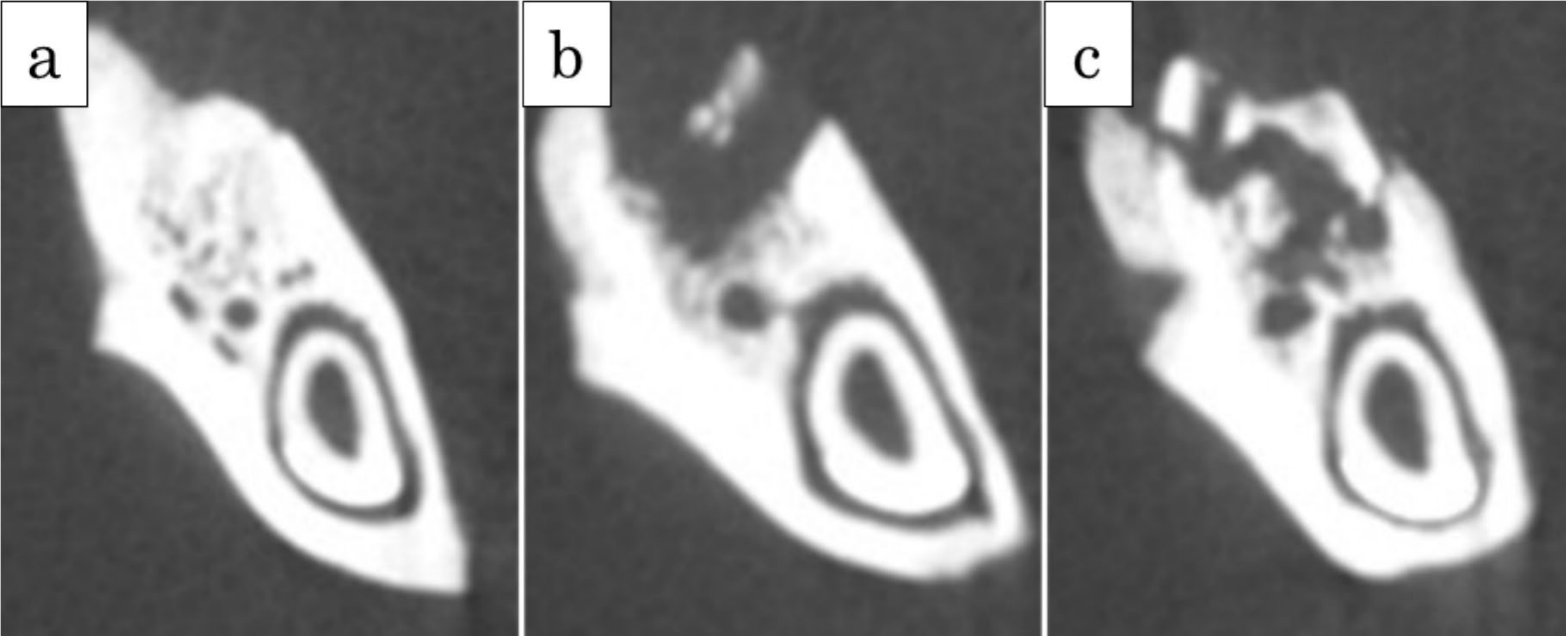
Typical micro-CT images. **a** Alveolar defect in the test group at 8 weeks after tooth extraction. There were no bone fragments, and new bone formation was found in the extraction socket (score 0). **b** Alveolar defect in the control group at 8 weeks after tooth extraction. There was sequestration of the alveolar bone in the extraction socket (score 1). **c** Alveolar defect in the control group at 8 weeks after tooth extraction. There were bone fragments suggestive of bone sequestration in the entire alveolar ridge (score 2)

Micro-CT analyses revealed evidence of new bone formation within the extraction sockets of the test group (Fig. 3a). Conversely, the control group displayed alveolar bone sequestration (Fig. 3b) and bone fragments indicative of bone sequestration along the alveolar ridge (Fig. 3c).

In the test group, the distribution was as follows: 66.7% (8/12) scored 0, 25% (3/12) scored 1, and 8.3% (1/12) scored 2. For the control group, 8.3% (1/12) were categorized as score 0, 16.7% (2/12) as score 1, and 75% (9/12) as score 2. There was a statistically significant increase in the instances of score 2 in the control group compared to the test group (*P* < 0.001) (Table 1).

**Table 1:** The incidence of bone fragments in the extraction sockets

Score	Definition to description	Number	
		test (*n* = 12)	Control (*n* = 12)
0	No sequestration	8 (66.7%)	1 (8.3%)
1	Partial sequestration of the alveolar bone	3 (25%)	2 (16.7%)
2	Sequestration of the entire alveolar ridge	1 (8.3%)	9 (75%)

Micro-CT images showed new bone formation in the extraction sockets in the test group (Fig. 3a). On the other hand, there was sequestration of the alveolar bone (Fig. 3b) and bone fragments suggestive of bone sequestration in the entire alveolar ridge (Fig. 3c) in the control group.

In the test group, 66.7% (8/12), 25% (3/12), and 8.3% (1/12) were classified as score 0, score 1, and score 2, respectively. In the control group, 8.3% (1/12), 16.7% (2/12), and 75% (9/12) were classified as score 0, score 1, and score 2, respectively. The number of score2 was significantly higher in the control group than in the test group (*P* < 0.001; Table 1).

### Quantification of new bone formation

**Fig. 4:**
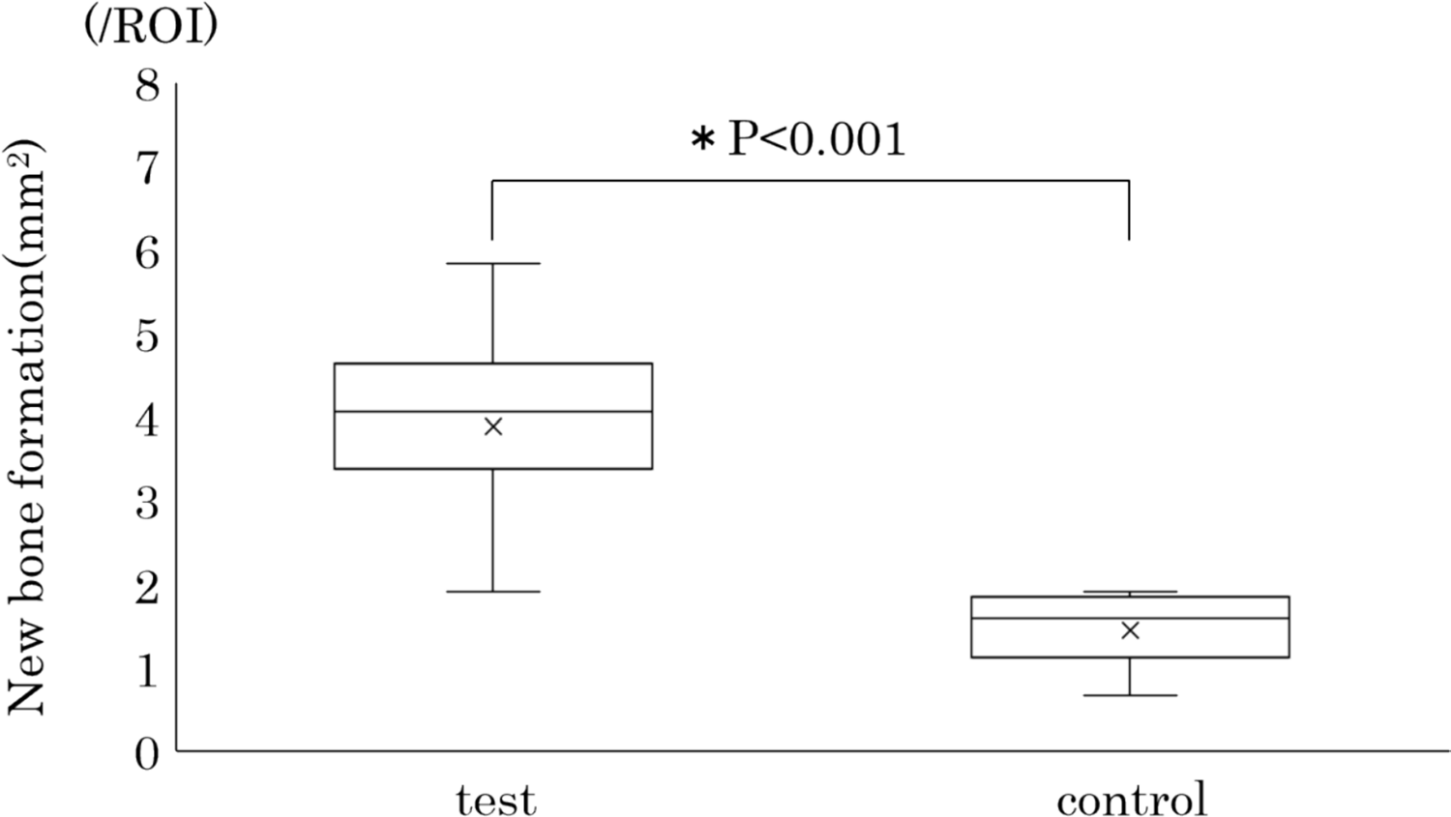
The proportion of the new bone formation in the extraction socket. Quantifying the area of new bone formation revealed a significantly positive volume of new bone formation in the test group compared to that in the control group (*P* < 0.001)

The new bone formation area was 3.88 ± 1.06 mm^2^ in the test group and 1.45 ± 0.40 mm^2^ in the control group. Significantly larger areas of new bone formation were noted within the extraction socket in the test group compared to the control group (*P* < 0.001; Fig. 4).

### Histological findings

**Fig. 5:**
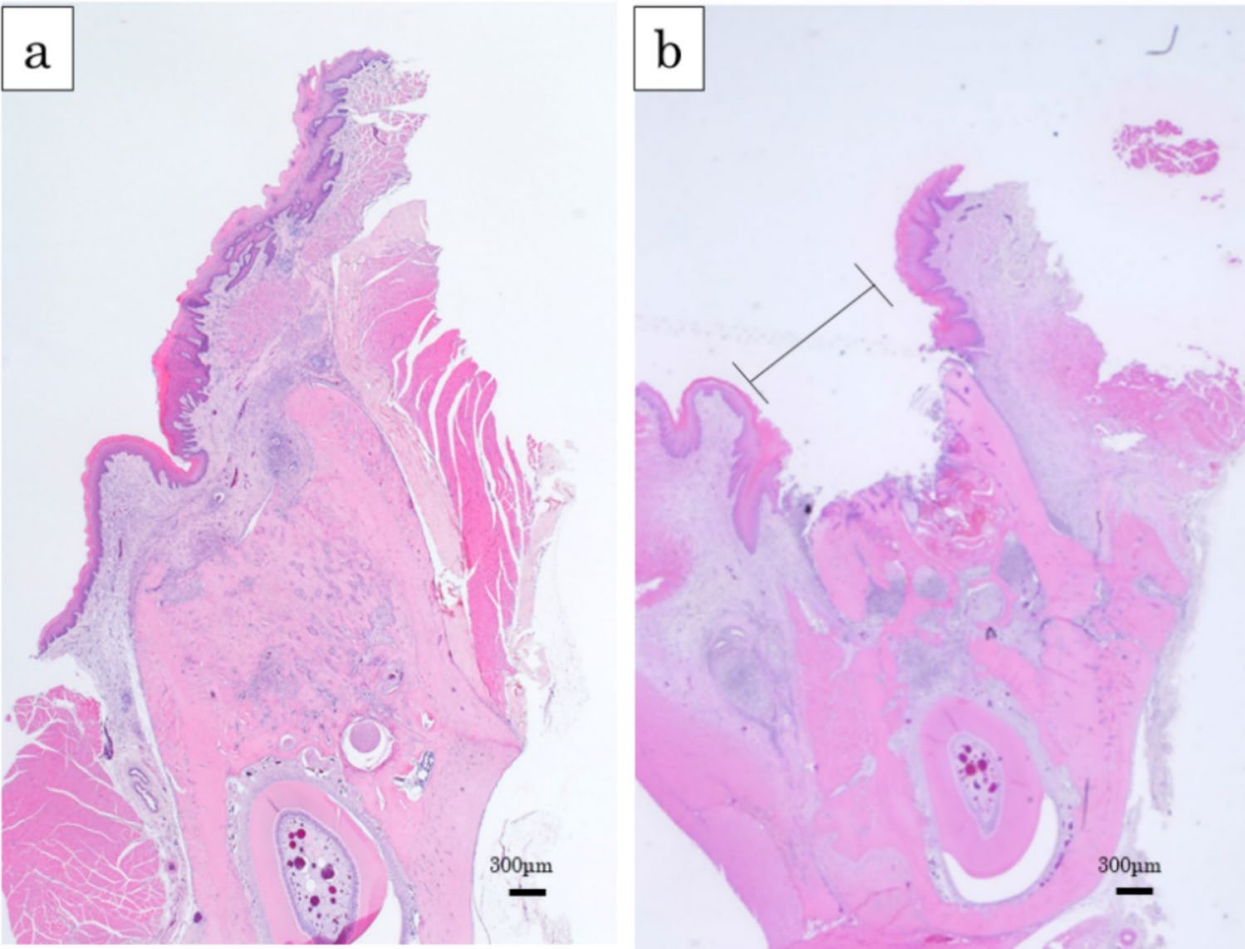
Typical histological findings. **a** Alveolar defect in the test group at 8 weeks after tooth extraction. Mucosal disruption was not present. The defect was filled with new bone. **b** Alveolar defect in the control group at 8 weeks after tooth extraction. Mucosal disruption (black line) was present

Mucosal disruption or sequestration in the extraction sockets was absent in the test group (Fig. 5a) but present in the control group (Fig. 5b). The presence or absence of mucosal disruption and sequestration in the extraction sockets were evaluated at 8 weeks after tooth extraction (Table 2). Mucosal disruption and sequestration were significantly higher in the control group than in the test group (100% [12/12] vs. 8.3% [1/12] *P* < 0.001; 100% [12/12] vs. 33.3% [4/12] *P* < 0.001; respectively).

**Table 2:** The incidence of mucosal disruption and sequestration

	Number	
	test (*n* = 12)	Control (*n* = 12)
Mucosal disruption	1 (8.3%)	12 (100%)
Sequestration	4 (33.3%)	12 (100%)

### Immunohistochemical findings

**Fig. 6:**
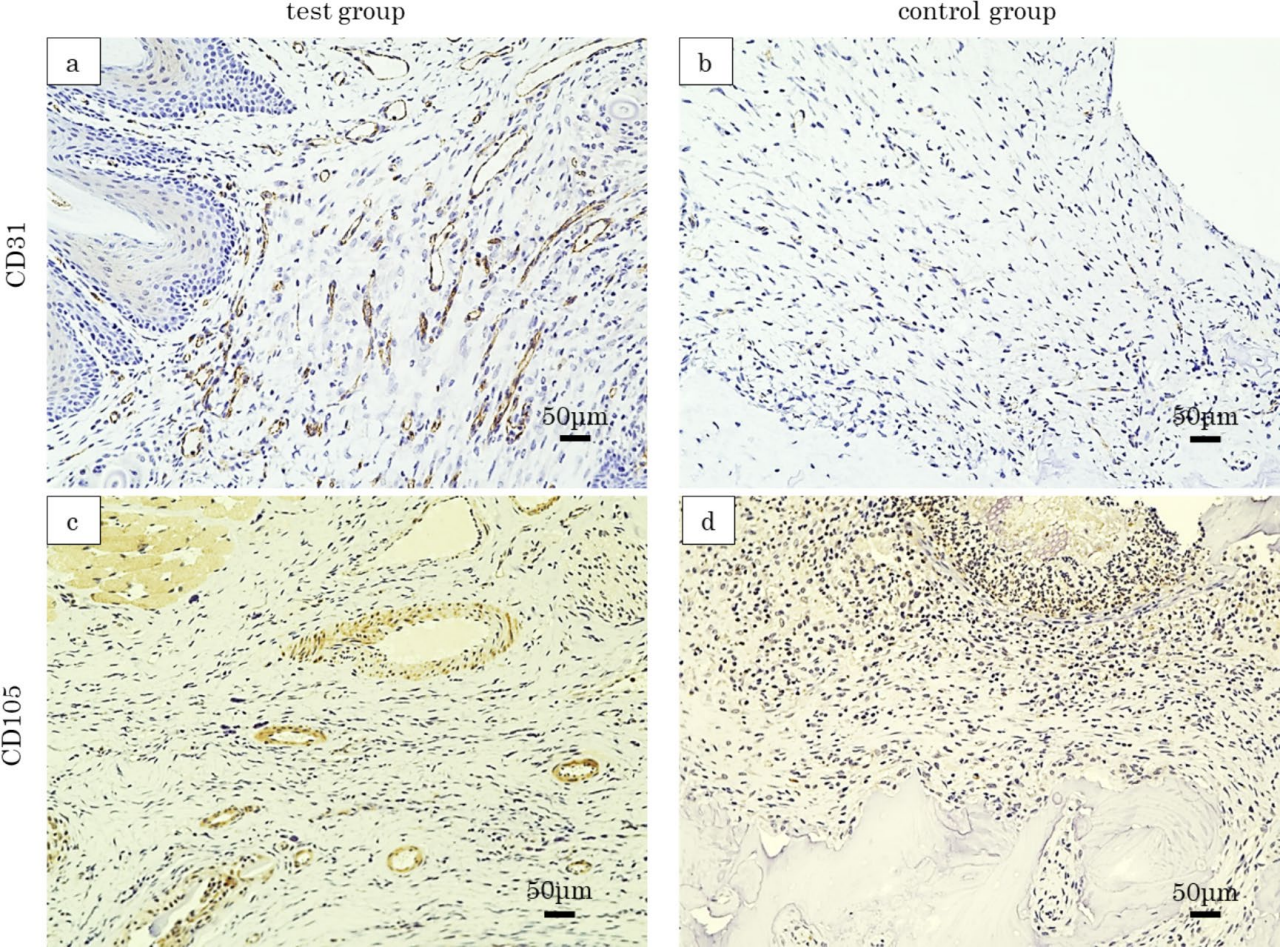
Typical immunohistochemical findings. **a** CD31 vascular staining in the test group. Numerous CD31-positive vessels were observed in the lamina propria of the extraction socket. **b** CD31 vascular staining in the control group. Only a sparse presence of CD31-positive vessels was noted. **c** CD105 vascular staining in the test group. A number of CD105-positive vessels were present in the lamina propria of the extraction socket. **d** CD105 vascular staining in the control group. CD105-positive vessels were scarcely detected

**Fig. 7:**
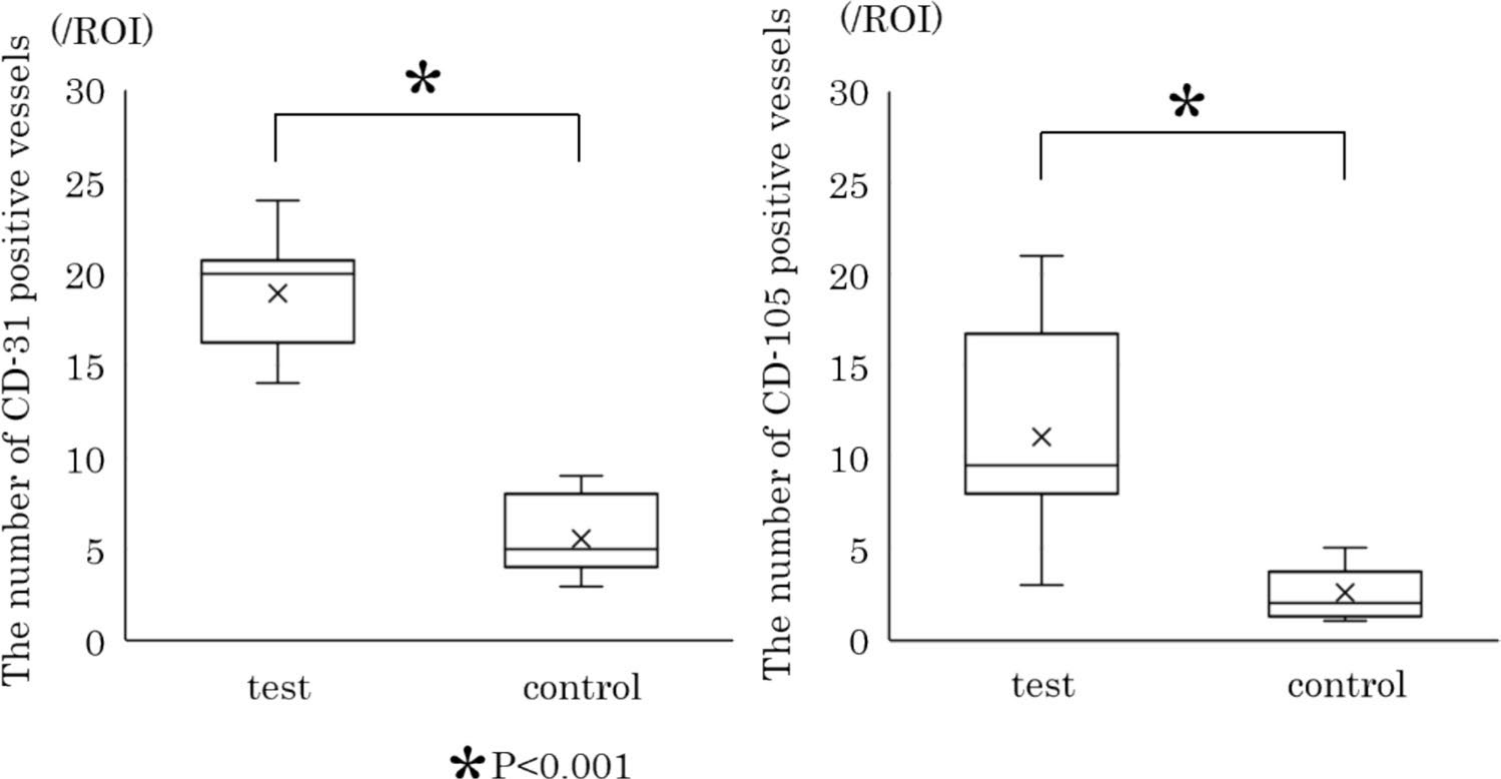
The number of CD-31 and CD-105 positive vessels in the sub-epithelial lamina propria. The numbers of sub-epithelial CD31- and CD105-positive vessels in the test group were higher than those in the control group (*P* < 0.001)

In the test group, a multitude of CD31-positive vessels can be observed within the lamina propria of the extraction socket (Fig. 6a), whereas in the control group, CD31-positive vessels are scarcely identified (Fig. 6b). Similarly, for CD105 immunostaining, the test group exhibits several positive vessels within the lamina propria of the extraction socket (Fig. 6c), but in the control group, positive vessels are almost undetectable (Fig. 6d). The numbers of CD31- and CD105-positive vessels were significantly higher in the test group than in the control group (18.9 ± 3.1 vs. 5.6 ± 2.0, *P* < 0.001; 11.1 ± 5.2 vs. 2.6 ± 1.4, *P* < 0.001; respectively; Fig. 7).

## Discussion

Efforts to prevent BRONJ and to promote bone regeneration have been documented [27–31]; however, the translation of these findings into clinical practice remains limited, underscoring the necessity for novel preventative strategies. Our investigation centered on whether the local administration of basic fibroblast growth factor (bFGF) in extraction sockets could reduce the incidence of BRONJ post-extraction. Remarkably, our study revealed that 8 weeks following tooth extraction, BRONJ occurred in 100% of the control group (12/12), in stark contrast to the test group, which exhibited a reduced incidence of 8.3% (1/12). This substantial difference suggests that bFGF’s local application is a viable strategy for BRONJ prevention. Given these encouraging results, the application of bFGF could be beneficial in high-risk BRONJ patients undergoing periodontal or oral surgery. However, further clinical trials are necessary to establish optimal protocols, including timing, dosing, and application methods, before it can be widely recommended for routine clinical practice.

In this study, HPC, a non-reactive cellulose derivative, was used to increase the viscosity of bFGF solutions without altering their bioactivity, capitalizing on its tendency to form a viscous gel upon hydration [17]. We focused on the pro-angiogenic properties of bFGF, given the critical role of mucosal healing and neovascularization in BRONJ prevention post-extraction [11, 12, 30]. CD31 and CD105, endothelial and mesenchymal stem cell markers, respectively, have been shown to be less expressed in BRONJ-affected mucosa [32, 33]. Our study indicated an increase in these markers in the test group, suggesting bFGF’s role in offsetting the anti-angiogenic effects of BPs.

The clinical application of bFGF, known for its angiogenic efficacy, includes treatments for skin ulcers and tympanic membrane perforations [20–23]. In Japan, a bFGF formulation, RegrothⓇ, was approved for alveolar bone loss from periodontitis in 2016 [24], containing the same concentration of bFGF and HPC as our experimental setup [24]. This similarity suggests potential drug repositioning opportunities for RegrothⓇ in preventing BRONJ.

It is important to note that while the ZA dosage in our study was equivalent to human use, the timing was deliberately closer to the surgical procedure to induce BRONJ within the experiment’s timeframe. This approach differs from clinical practice, where BP administration and surgery are spaced apart to minimize complications. Although this accelerated timing effectively modeled BRONJ in rats, future studies should consider timing adjustments to better reflect clinical conditions.

In addition, combining bFGF with alveolar bone graft materials may enhance bone regeneration and minimize post-extraction concavity due to alveolar bone resorption, while also improving bFGF retention and application for better outcomes. However, ensuring hydrogel retention in the extraction socket remains challenging. While our study employed a closure technique, complete closure is often difficult in clinical practice. To address this, we propose using a gelatin sponge, such as Spongel® or Gelfoam®, following Regroth® application. This method could help retain the hydrogel within the socket [23], though additional measures, such as suturing or applying fibrin glue, may still be necessary.

In conclusion, our study’s findings with a RegrothⓇ-like application suggest a promising approach for BRONJ prevention. This could lead to a novel therapeutic strategy for patients at risk of developing BRONJ post-tooth extraction. Further research into the optimal application protocols and potential combination therapies is warranted.

## Data Availability

All data included in this study are available upon request by contact with the corresponding author.
